# Integrated transcriptomics and proteomics analysis reveals muscle metabolism effects of dietary *Ulva lactuca* and ulvan lyase supplementation in weaned piglets

**DOI:** 10.1038/s41598-024-55462-2

**Published:** 2024-02-26

**Authors:** David Miguel Ribeiro, Diogo Coelho, Mónica Costa, Daniela Filipa Pires Carvalho, Céline C. Leclercq, Jenny Renaut, João Pedro Bengala Freire, André Martinho Almeida, José António Mestre Prates

**Affiliations:** 1 Associate Laboratory TERRA, LEAF - Linking Landscape, Environment, Agriculture and Food Research Centre, Instituto Superior de Agronomia, Universidade de Lisboa, Tapada da Ajuda, 1349-017 Lisbon, Portugal; 2 Faculdade de Medicina Veterinária, CIISA - Centre for Interdisciplinary Research in Animal Health, Universidade de Lisboa, 1300-477 Lisbon, Portugal; 3 Laboratório Associado para Ciência Animal e Veterinária (AL4AnimalS), Lisbon, Portugal; 4 Biotechnology Environmental Analysis Platform (BEAP), Environmental Research and Innovation Department (ERIN), LIST- Luxembourg Institute of Science and Technology, 5, Rue Bommel, 4940 Hautcharage, Luxembourg; 5 Centre of Molecular and Environmental Biology (CBMA), University of Minho, Campus de Gualtar, 4710-057 Braga, Portugal

**Keywords:** Weaned piglet, *Ulva lactuca*, Muscle, Transcriptomics, Proteomics, Proteomic analysis, Protein-protein interaction networks, Metabolism

## Abstract

Seaweeds, including the green *Ulva lactuca*, can potentially reduce competition between feed, food, and fuel. They can also contribute to the improved development of weaned piglets. However, their indigestible polysaccharides of the cell wall pose a challenge. This can be addressed through carbohydrase supplementation, such as the recombinant ulvan lyase. The objective of our study was to assess the muscle metabolism of weaned piglets fed with 7% *U. lactuca* and 0.01% ulvan lyase supplementation, using an integrated transcriptomics (RNA-seq) and proteomics (LC–MS) approach. Feeding piglets with seaweed and enzyme supplementation resulted in reduced macronutrient availability, leading to protein degradation through the proteasome (PSMD2), with resulting amino acids being utilized as an energy source (GOT2, IDH3B). Moreover, mineral element accumulation may have contributed to increased oxidative stress, evident from elevated levels of antioxidant proteins like catalase, as a response to maintaining tissue homeostasis. The upregulation of the gene AQP7, associated with the osmotic stress response, further supports these findings. Consequently, an increase in chaperone activity, including HSP90, was required to repair damaged proteins. Our results suggest that enzymatic supplementation may exacerbate the effects observed from feeding *U. lactuca* alone, potentially due to side effects of cell wall degradation during digestion.

## Introduction

The human population is expected to reach 10 billion by 2050^1^. Combined changes in consumer preferences and increased demand for animal products are expected to exert tremendous pressure on the livestock sector to increase production, whilst increasing the need for promoting environmental sustainability^2^. In particular, the demand for pork is expected to increase substantially^3^, thereby pressuring upstream production factors, such as energy and arable land towards the production of conventional feedstuffs, including maize and soybean meal^4^. In the European context, this causes a problem, where local, sustainable, and high-quality feedstuffs are necessary, to counteract the negative environmental and economic impacts of commodity imports. This is particularly important given the rising inflation rate, political instability, and conflict in Eastern Europe^5^.

Seaweeds can play a role in increasing the independence of European countries from external markets to satisfy internal demand for feedstuffs^6^. Indeed, the European seaweed industry is expected to grow steadily until at least 2030 according to some estimates, which could give rise to cost-effective products^7^. These organisms are highly heterogeneous and widely distributed, having particular nutritional properties such as a high ash content, healthy fatty acid profile and high concentrations of other bioactive compounds, including laminarin^8^ and ulvan^9^. Moreover, green seaweeds (*Chlorophyceae*) such as those of the *Ulva* genus have moderate levels of crude protein (CP) and energy that make it interesting to be included in animal diets, including pigs. When considering human diets, some authors have found that their essential amino acid composition surpasses that of conventional foodstuffs such as cereal grains (maize, wheat) and legumes (soybeans)^10^, however, this seems to depend on several factors including harvesting location and season. Indeed, other authors have reported that *U. lactuca* has virtually half the lysine content of soybean meal^11^. Regardless, they have high iron (Fe) and α-linolenic contents, which are beneficial for piglets and improve meat quality^4,11–13^. Despite this potential as a nutrient source for monogastric diets, to the best of our knowledge, the whole biomass of *Ulva* sp. has not been reported to be used for this purpose. However, extracts from these seaweeds have been reported to be used in feeding farm animals. Samarasinghe et al.^14^ have reported the effects of milk supplementation with *Ulva* sp. fed to preweaning calves on performance and gut health. In turn, Bussy et al.^15^ reported that sulphated polysaccharide extract of *U. armoricana* has immunomodulatory properties when fed to sows, for example, by increasing IgG concentrations in their milk. An extract with polyphenols and unsaturated fatty acids from *U. prolifera* is reported to increase total antioxidant capacity and superoxide dismutase (SOD) activity in the serum of weaned piglets challenged with hydrogen peroxide^16^. Recently, dietary *U. lactuca* has been reported as a feed ingredient in broiler chicken diets^17,18^. However, to the best of our knowledge, the use of whole *Ulva sp.* biomass as a feed ingredient (> 3% dietary incorporation) in piglet diets is scarcely studied^19^, possibly because of the antinutritional effects that can occur due to their highly complex cell wall polysaccharides^13^. This is partly caused in turn by the highly complex, cross-linked ulvan, a sulphated polysaccharide made up of rhamnose, glucuronic acid and xylose monomers^11^. In vitro tests have reported the effectiveness of a single ulvan lyase to disrupt the *U. lactuca* cell wall, showing promise for in vivo trials^20^. Using such an approach could allow the use of the whole biomass of the seaweed, avoiding the cost-increasing extraction and taking advantage of the full composition of the seaweed, including health-promoting iodine and n-3 PUFA, as well as polysaccharides with prebiotic properties.

In addition to the current lack of knowledge about dietary whole *U. lactuca* as a feed ingredient, its effect on metabolism is so far unknown. The advent of high-throughput molecular biology tools, Omics, such as transcriptomics and proteomics, allows having an in-depth look into tissue metabolism under the effect of a certain dietary factor^21,22^. The combined study of these often-large datasets is gaining track in animal science, with few examples so far in the particular case of the pig. For instance, Voillet et al.^23^ and Murgiano et al.^24^ have reported proteomic and transcriptomic results using gel-based proteomics and microarray transcriptomics techniques in the pig. Our team has recently demonstrated the effect of dietary *Chlorella vulgaris* on the muscle transcriptome and proteome of finishing pigs, using such technologies^25^. The objective of this work was to perform a hitherto not performed analysis of the weaned piglet muscle metabolism under the effect of 7% dietary *U. lactuca* (UL) and 0.01% ulvan lyase supplementation (ULU), using state-of-the-art and high-throughput transcriptomics and proteomics data.

## Results

### Growth performance and longissimus lumborum composition

These results have been previously published in companion papers and are briefly mentioned to provide context^19,26^. Diets had no statistically significant effect on the growth performance of piglets. The average final live weights of control, UL and ULU piglets were 14.7, 15.0 and 14.9 kg, respectively. Meat quality parameters such as colour (L*, a* and b*), cooking loss and shear force, were not affected by dietary treatments. In turn, some sensorial attributes (*e.g.* tenderness, juiciness) were improved in ULU compared to either control or UL.

### Transcriptomics analysis

The total number of reads generated by RNA-seq, as well as identified genes, is reported in Supplementary Table S1. The average number of raw reads obtained per experimental group was 36,750,790, 39,468,426, and 41,358,016 for control, UL and ULU, respectively. After trimming, an average of 35,282,799 (96%), 37,978,048 (96%) and 40,132,353 (97%) reads remained for control, UL and ULU, respectively. These were then mapped on the *Sus scrofa* genome, yielding an average of 32,459,277 (92%), 34,979,791 (92%) and 37,287,649 (93%) reads for control, UL and ULU, respectively. The most abundant genes, ranked from highest (1,608,943) to lowest (290,438) average counts regardless of group, were: actin alpha 1 (*ACTA1*), myosin light chain 1 (*MYL1*), myosin light chain 11 (*MYL11*), myosin 2 (*MYH2*), a mitochondrial rRNA (ENSSSCG00000018063), creatine kinase, M-type (*CKM*), lactate dehydrogenase A (*LDHA*), tropomyosin 1 (*TPM1*), a long non-coding RNA (ENSSSCG00000048719) and NADH:ubiquinone oxidoreductase core subunit 4 (*ND4*).

**Figure 1:**
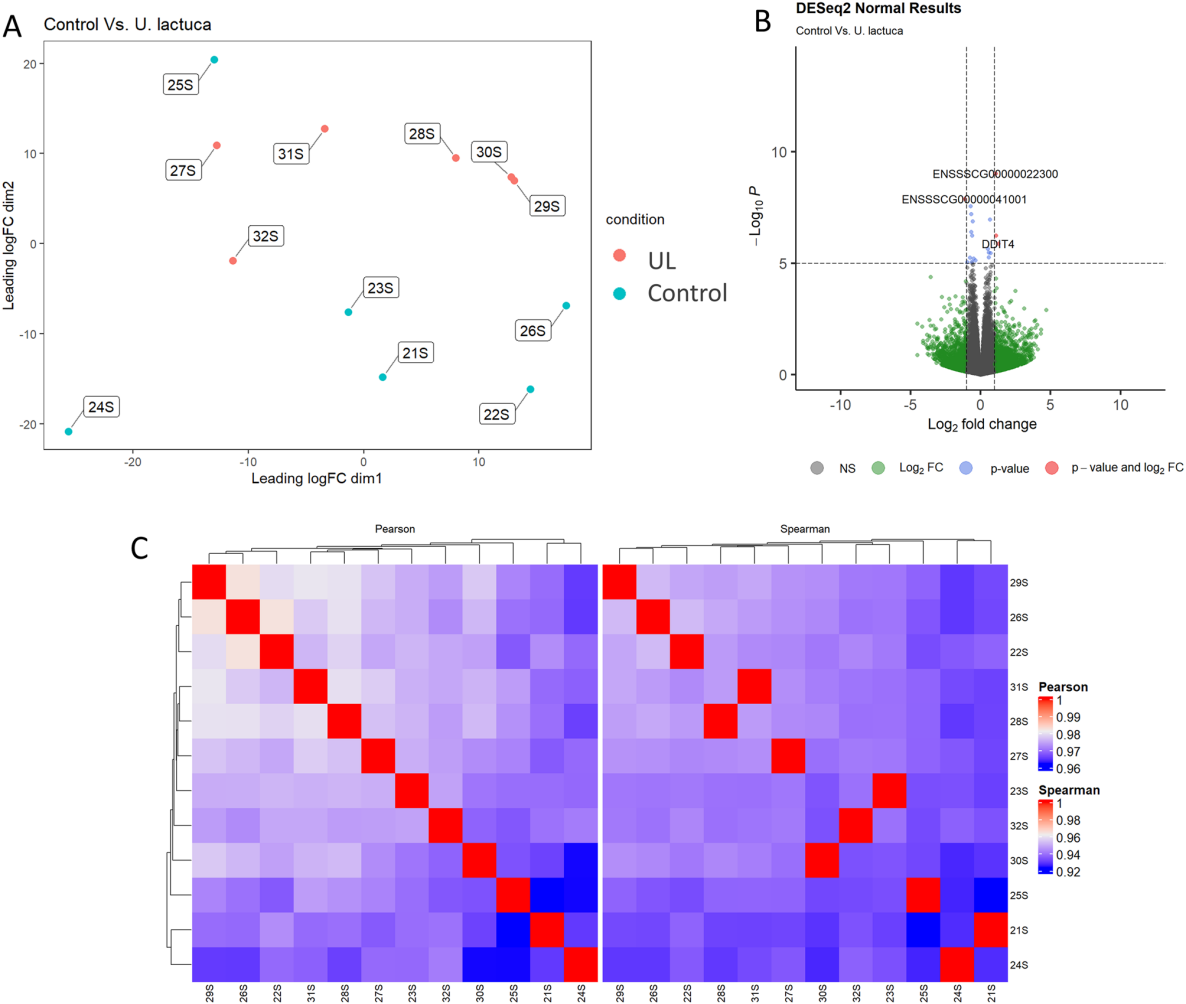
(**A**) Multi-dimensional scaling (MDS) plot showing the relationship between control (wheat, maize and soybean meal-based diet) and UL (7% *Ulva lactuca* replacing control) samples. (**B**) Volcano plot depicting the − log_10_(Pvalue) and log_2_(Fold change), as evidence of differential gene expression. (**C**) Clustering of sample-to-sample correlations using Pearson (left) or Spearman (right) correlations.

The differential gene expression analysis revealed 11, 20 and 4 differentially expressed genes (DEGs) in UL vs control, ULU vs control and ULU vs UL (Supplementary Figure S1). The first comparison, UL vs control, was characterized by an overall downregulation and upregulation of 8 and 3 genes, respectively, in relationship to control animals (Fig. 1). The downregulated genes were: Nuclear Receptor Subfamily 4 Group A Member 3 (*NR4A3,* log_2_(fold change) = − 2.48), Activating Transcription Factor 3 (*ATF3,* log_2_(fold change) = − 2.18), ChaC Glutathione Specific Gamma-Glutamylcyclotransferase 1 (*CHAC1,* log_2_(fold change) = − 2.08), DNA Damage Inducible Transcript 4 (*DDIT4,* log_2_(fold change) = − 1.28), LOC110255260 (log_2_(fold change) = − 1.14), Methionine Adenosyltransferase 2A (*MAT2A,* log_2_(fold change) = − 1.08), Interleukin 33 (*IL33,* log_2_(fold change) = − 1.07) and Doublecortin Domain Containing 1 (*DCDC1,* log_2_(fold change) = − 1.00). The upregulated genes were a long non-coding RNA (LncRNA, log_2_(fold change) = 1.12), ribosomal RNA (*RN5-8S,* log_2_(fold change) = 1.39) and Cytokine Inducible SH2 Containing Protein (*CISH,* log_2_(fold change) = 3.57). The affected biological processes include positive regulation of vascular smooth muscle cell proliferation (GO:1904707), gluconeogenesis (GO:0006094), and intracellular signal transduction (GO:0035556).

**Figure 2:**
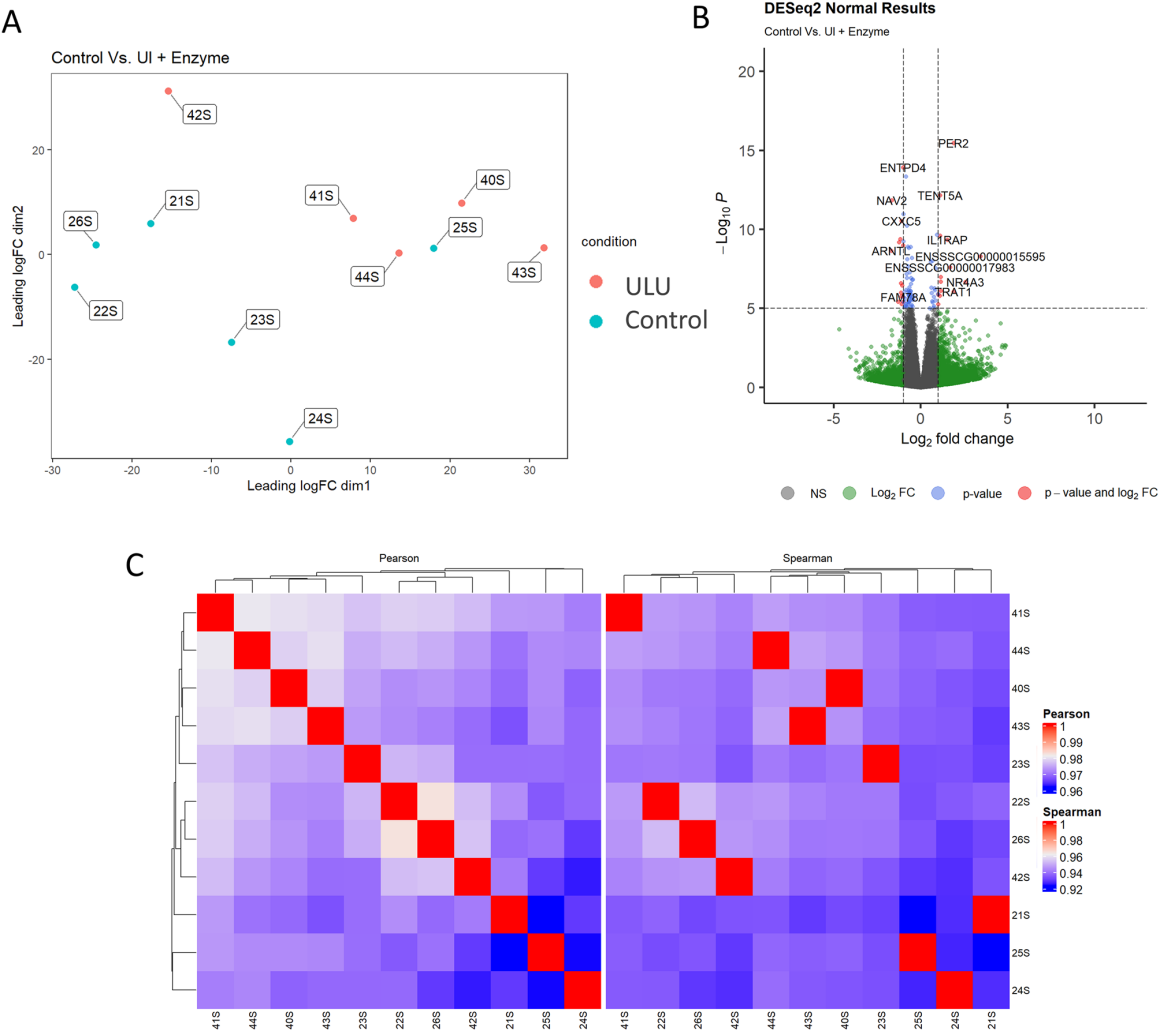
(**A**) Multi-dimensional scaling (MDS) plot showing the relationship between control (wheat, maize and soybean meal-based diet) and ULU (7% *Ulva lactuca* replacing control + 0.01% ulvan lyase) samples. (**B**) Volcano plot depicting the − log_10_(Pvalue) and log_2_(Fold change), as evidence of differential gene expression. (**C**) Clustering of sample-to-sample correlations using Pearson (left) or Spearman (right) correlations.

The second comparison, ULU vs control, resulted in the highest number of DEGs, with 10 being up and 10 being downregulated (Fig. 2). The 3 most upregulated genes in ULU were aryl hydrocarbon receptor nuclear translocator like (*ARNTL,* log_2_(fold change) = 1.70), neuron navigator 2 (*NAV2,* log_2_(fold change) = 1.67) and troponin T1 (*TNNT1,* log_2_(fold change) = 1.30), with biological processes that include positive regulation of transcription from RNA polymerase II promoter (GO:0045944), neurogenesis (GO:0022008) and regulation of muscle contraction (GO:0006937), respectively. In turn, the three most downregulated in ULU were *ATF3* (log_2_(fold change) = − 3.44), *NRAA3* (log_2_(fold change) = − 2.57) and *CHAC1* (log_2_(fold change) = − 2.45), like what occurred in the previous comparison for UL.

**Figure 3:**
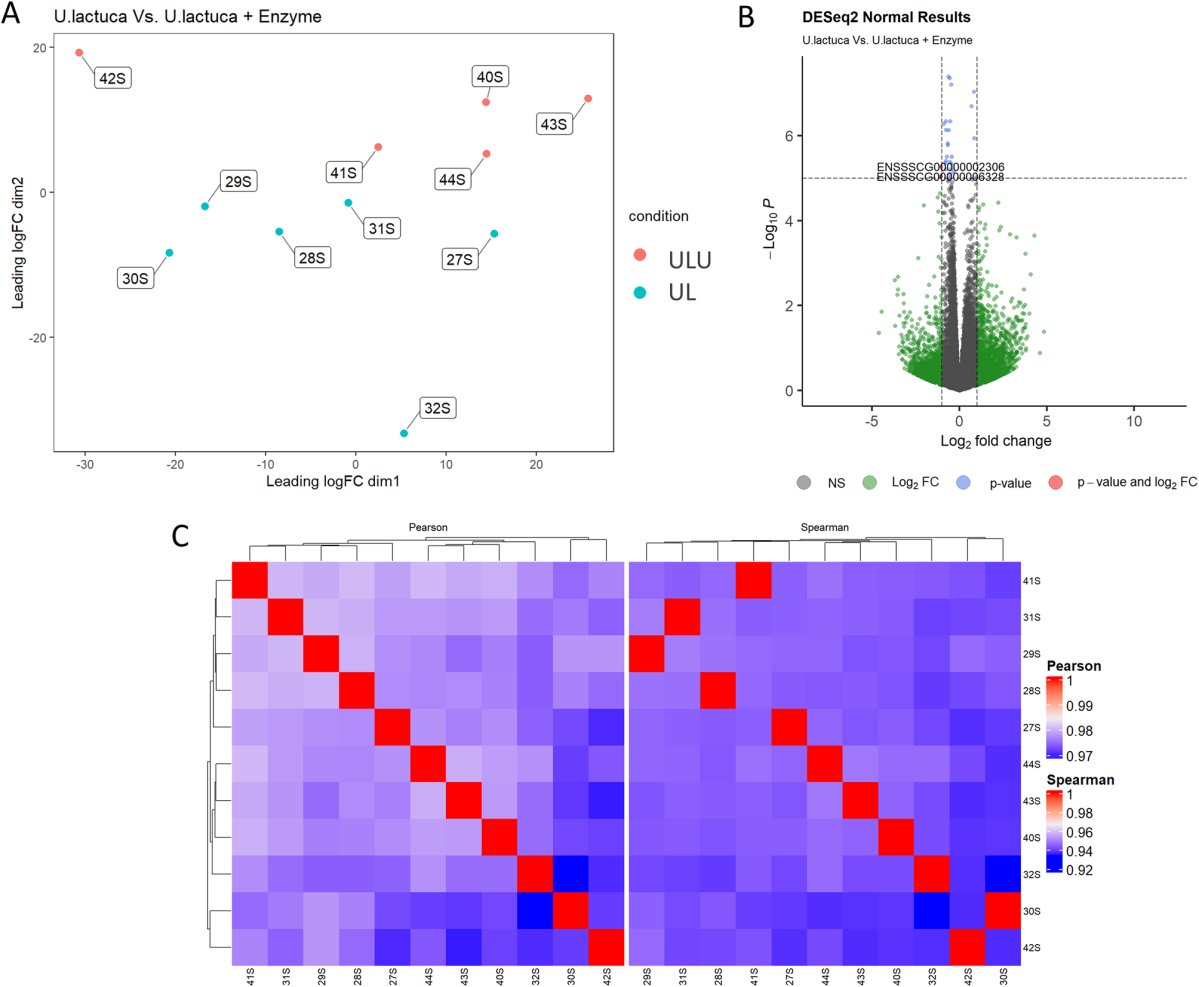
(**A**) Multi-dimensional scaling (MDS) plot showing the relationship between UL (7% *Ulva lactuca* replacing control) and ULU (UL + 0.01% ulvan lyase) samples. (**B**) Volcano plot depicting the − log_10_(Pvalue) and log_2_(Fold change), as evidence of differential gene expression. (**C**) Clustering of sample-to-sample correlations using Pearson (left) or Spearman (right) correlations.

Comparing both seaweed groups resulted in the lowest number of DEGs (Fig. 3). The two upregulated genes in ULU were smoothelin-like 1 (*SMTNL1,* log_2_(fold change) = 1.03) and retinoid X receptor gamma (*RXRG,* log_2_(fold change) = 1.09), which participate in actin cytoskeleton organization (GO:0030036) and steroid hormone-mediated signalling pathway (GO:0043401), respectively. The downregulated ones were a heparin-binding-like growth factor (*HBEGF,* log_2_(fold change) = − 1.35) and a mitochondrial RNA (Mt rRNA, log_2_(fold change) = − 1.09), the former participating in the positive regulation of smooth muscle cell proliferation (GO:0048661).

### Proteomics analysis

**Figure 4:**
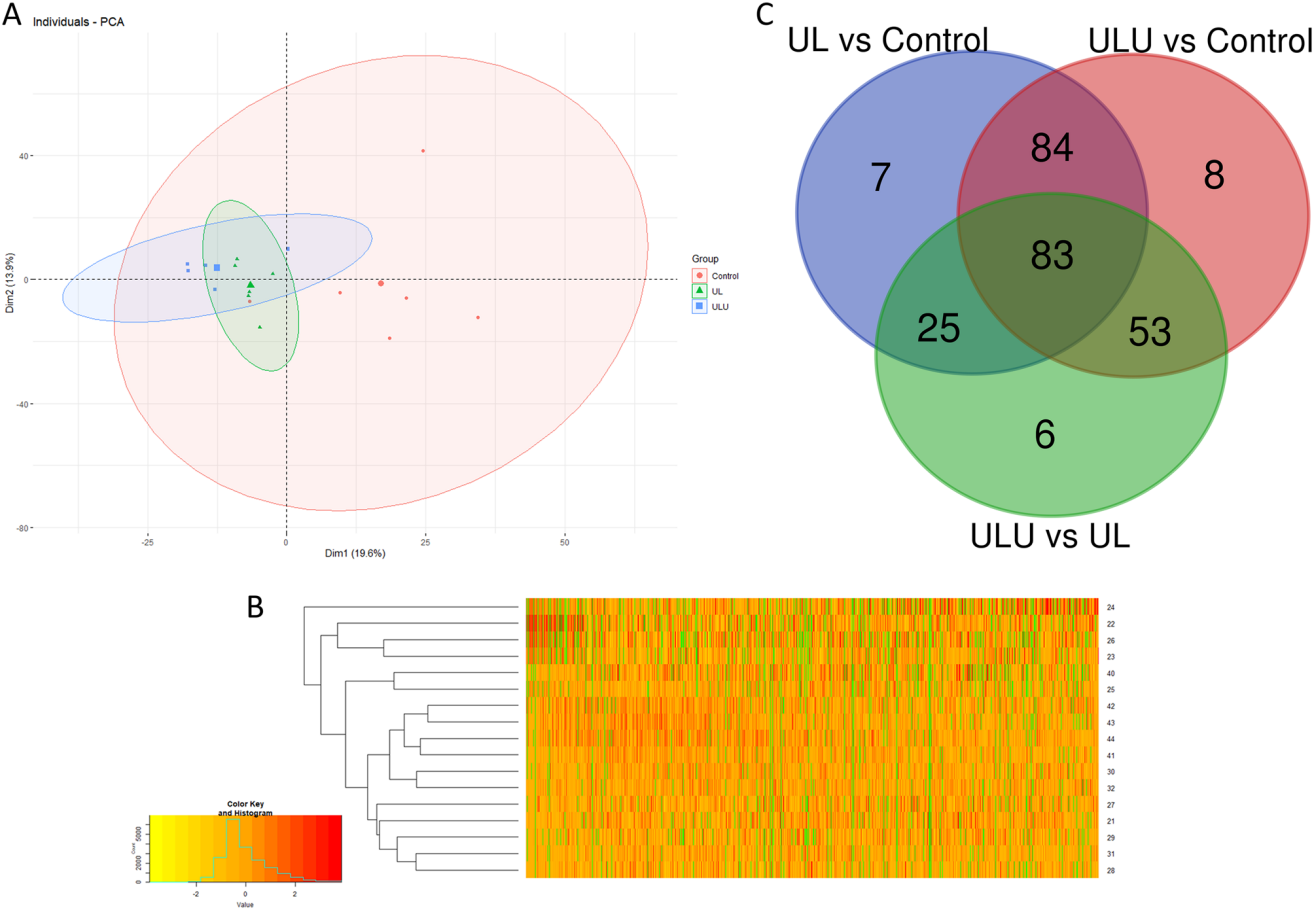
(**A**) Principal component analysis on the *longissimus lumborum* proteome, (**B**) Heatmap of the *longissimus lumborum* proteome (green is NA values), and (**C**) Venn diagram of differentially abundant proteins between three comparisons of piglets fed with control (wheat, maize, soybean meal-based diet), UL (7% *Ulva lactuca* replacing control) and ULU (UL + 0.01% ulvan lyase).

In this study, we identified 1208 proteins in the *longissimus lumborum* muscle of piglets (Supplementary Table S2), with at least 2 identified peptides and at least 1 unique peptide. From the multivariate analysis carried out on the identified proteins, the control group was more heterogeneous than either UL or ULU (Fig. 4A), and there was not a distinct clustering of any experimental group (Fig. 4B). From them, a total of 266 were found to be differentially abundant proteins (DAPs), with *P*-value < 0.05 and fold change < 0.67 or fold change > 1.5. We calculated pairwise comparisons and found that of these, 199, 228 and 167 DAPs were different between UL vs control, ULU vs control and ULU vs UL, respectively (Fig. 4C).

**Figure 5:**
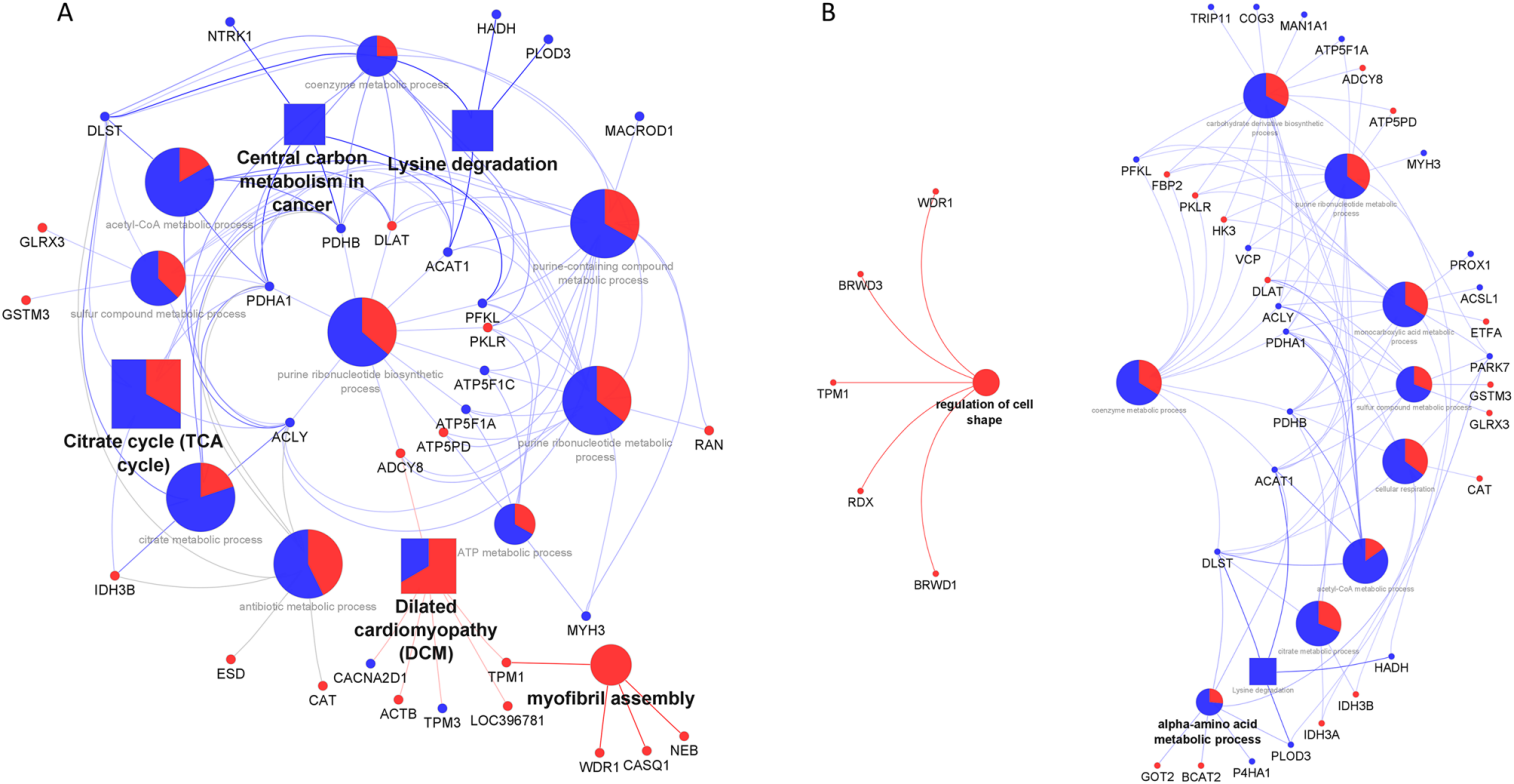
Functional analysis output obtained by the ClueGO tool in Cystoscape, for the proteomic comparisons of UL vs Control (**A**) and ULU vs Control (**B**). Red-UL and ULU (in **A** and **B**, respectively); Blue—Control. Circles—Biological Process; Squares—KEGG pathways. The fill colour percentage is according to the number of proteins connected to the pathway from each group.

The functional analysis for the DAPs of the UL vs control comparison is presented in Fig. 5A. Significant term *P*-values corrected with Bonferroni are presented after each named term identified in the analysis. The control group was the only one that had DAPs connected to Central carbon metabolism in cancer (*P*-value = 0.0174; NTRK1, PDHA1, PDHB, PFKL) and Lysine degradation (*P*-value = 0.0163; ACAT1, DLST, HADH, PLOD3); whereas the seaweed group increased the abundance of proteins that were related with myofibril assembly (*P*-value = 0.0143; CASQ1, NEB, TPM1, WDR1). The remaining pathways were all detected with a predominance of control proteins, such as the Citrate cycle (TCA cycle) (*P*-value < 0.0001; ACLY, DLST, PDHA1, PDHB), except Dilater cardiomyopathy (DCM) (*P*-value = 0.0006; ACTB, ADCY8, LOC396781, TPM1).

The functional analysis for the DAPs for the ULU vs control comparison is shown in Fig. 5B. Similarly to the last comparison, the control group had most of the identified proteins linked to most of the pathway terms, except regulation of cell shape (*P*-value = 0.0471; BRWD1, BRWD3, RDX, TPM1, WDR1) which was found only about the ULU proteins. Conversely, lysine degradation only corresponded to control DAPs (*P* = 0.0412; ACAT1, DLST, HADH, PLOD3). One particular pathway, coenzyme metabolic process (*P*-value = 0.0003), revealed a connection with several ULU and control DAPs that are in turn related to pathways such as carbohydrate derivative biosynthetic process (*P*-value < 0.0001) and alpha-amino acid metabolic process (*P*-value = 0.0150).

**Figure 6:**
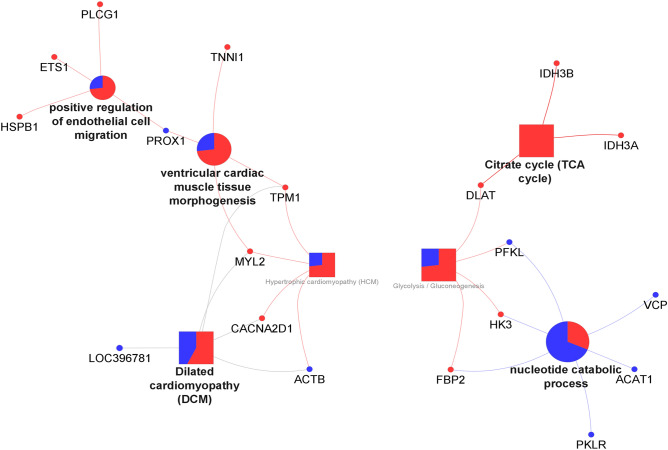
Functional analysis output obtained by the ClueGO tool in Cystoscape, for the proteomic comparison of ULU vs UL. Red—ULU; Blue—Control. Circles—Biological Process; Squares—KEGG pathways. The fill colour percentage is according to the number of proteins connected to the pathway from each group.

The functional analysis for the DAPs for the ULU vs UL comparison is presented in Fig. 6. This comparison yielded the lowest number of DAPs and also the least complex protein interaction network. In this comparison, ULU piglets had an exclusively higher abundance of proteins related to the citrate cycle (TCA cycle) pathway term (*P*-value = 0.0048; DLAT, IDH3A, IDH3B), which was connected with nucleotide catabolic process (*P*-value = 0.0001) through glycolysis/gluconeogenesis (*P*-value = 0.0036) term.

### Data integration

**Figure 7:**
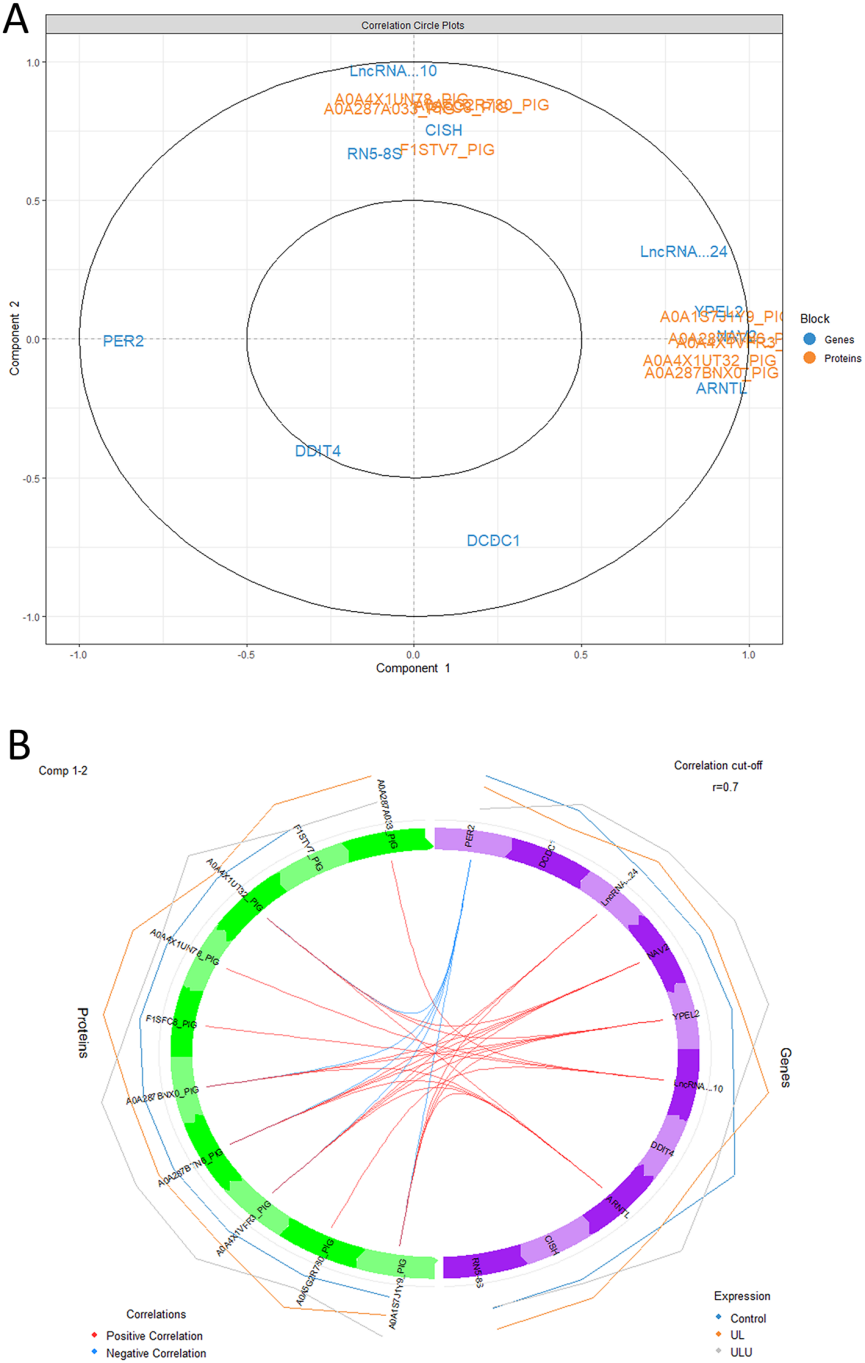
Correlation circle plot (**A**) and circos plot (**B**) for the differentially abundant proteins and differentially expressed genes found in the *longissimus lumborum* muscle of piglets fed with control (maize, wheat and soybean meal–based diet), UL (7% *Ulva lactuca* replacing control) and ULU (UL + 0.01% ulvan lyase).

The differentially abundant proteins and differentially abundant genes identified in the *longissimus lumborum* muscle of the piglets were used as input for integration using the DIABLO method of the *mixOmics* package in the RStudio environment. Both the correlation circle plot (A) and the circos plot (B) obtained are presented in Fig. 7. One gene, *PER2*, which was downregulated in ULU vs control, was negatively correlated with several proteins: A0A4X1UT32_PIG (aspartate aminotransferase, GOT2), A0A1S7J1Y9_PIG (Alpha2 chain of type I collagen, COL1A2), A0A287BNX0_PIG (Interferon regulatory factor 8, IRF8), A0A4X1VFR3_PIG (Diacylglycerol kinase, DGKH) and A0A287BTN6_PIG (Low-density lipoprotein receptor-related protein, LRP5). These proteins were all highly abundant in ULU vs control. The three genes *NAV2*, *YPEL2*, and *ARNTL* were all positively related to these proteins. They were found to be highly expressed in ULU vs control.

## Discussion

In this study, we conducted an integrated transcriptomics and proteomics analysis to investigate the effects of feeding *U. lactuca* and ulvan lyase supplementation on the muscle metabolism of weaned piglets. Our findings provide valuable insights into the molecular mechanisms underlying the utilization of seaweed as a feed ingredient and the potential impact of enzyme supplementation. To our knowledge, this is the first study with this purpose, other than the similar work we recently published for finishing pigs fed with *Chlorella vulgaris*^25^. In this case, samples were originated from piglets housed individually in metabolic cages in the framework of a digestibility assessment trial.

Comparing UL with control, it is evident that the latter group upregulated genes involved in similar, yet contradicting functions: lipolysis and fatty acid oxidation—*NR4A3*^27^, adipocyte differentiation—*MAT2A*^28^, muscle cell differentiation—*ATF3*^29^ and cell death—*DDIT4*^30^. This could reflect the theoretically increased digestive availability of nutrients of this diet, compared to the *U. lactuca* diet. Indeed, this is similar to what has been reported by Skugor et al.^27^, who identified a mobilization of endogenous nutrients in pigs fed with rapeseed meal (vs soybean meal) due to higher dietary fibre contents. In our study, control pigs demonstrate a putative increase in, for instance, protein turnover. This is similar to what we have found in our recent study on the use of *Chlorella vulgaris* to feed finishing pigs^25^, where microalgae-fed pigs increased the utilization of endogenous nutrients when compared to controls. This could be corroborated by our proteomics functional analysis, where we found, for instance, a higher abundance of HADH, PLOD3 and ACAT1 (lysine degradation) and PFKL, NTRK1, PDHB and PDHA1 (central carbon metabolism in cancer) in control piglets compared to UL. Both HADH and ACAT1 have been reported to be highly abundant in the *longissimus dorsi* of Chinese breed pigs (higher lipid deposition and lower muscle deposition when compared to western breeds), using an iTRAQ-based approach^31^. Accordingly, Serão et al.^32^ reported a positive correlation between PDHB (a pyruvate dehydrogenase) gene expression and intramuscular fat. Therefore, in addition to reflecting the higher nutrient availability inherent to the control diet, it is also apparent that lipid synthesis is higher in these pigs. This could furthermore be related to the fatty acid profile of the UL diet. Seaweeds are generally known to have a beneficial n-3 PUFA content^6^. In our study, we found that 18:4n-3 (stearidonic acid) was virtually absent in the control diet, as shown in our companion paper^19^. Corominas et al.^33^ have reported that tissue PUFA has an inverse relationship with lipogenic gene expression, like *SCD*, which could help explain the differences we found. However, lower muscle lipogenic gene expression has been reported to occur due to lower availability of glucose uptake in microalgae diets^25^, which might have happened in the current study. Lastly, the piglets fed with the UL diet upregulated the expression of *CISH*, which has been related to the high growth rate of Pietrain pigs^34^. Coherently, these same piglets had an increased abundance of contractile apparatus proteins, such as TPM1, WDR1, CASQ1 and NEB (myofibril assembly), as shown in our functional analysis. At first glance, this result could point to higher muscle development, i.e. growth rates, in UL piglets. However, control piglets comparatively increased the abundance of similar proteins, such as TPM3. Indeed, these piglets had no differences regarding their growth performance, which were minimized due to the allocation of piglets in a highly controlled environment, *e.g.* metabolic cages. Therefore, these particular differences likely lack biological implications.

Ulvan lyase supplementation increased the number of DEG’s compared to UL. Control piglets had an increased expression of *ATF3* and *NR4A3*, pointing to increased muscle cell differentiation and fatty acid oxidation, respectively. The scenario of seemingly contradictory gene functions repeats similarly to what was previously mentioned for the UL vs control comparison. In this comparison, control piglets upregulated *SESN2* and *ANPEP*, which participate in stress response and inhibit mTOR-mediated cell growth^35^, and have proteolytic activity^36^, respectively. In turn, they upregulated a key gene in circadian rhythm regulation of energy homeostasis and lipid metabolism^37^, *PER2*, as well as *ANKRD1*, involved in muscle tissue development^38^ and *ARG2*, which has been suggested to be an adequate biomarker for feed efficiency^39^. Their counterparts increased the abundance of *TNNT1*, which codes for a contractile apparatus protein that has been related to muscle growth^40^. Therefore, these differences do not seem to reflect different growth rates, but possibly differential nutrient availability. Indeed, control piglets have a theoretically higher nutrient availability, which is seemingly reflected in this differential gene expression, but also on the proteome level. They increased the abundance of pyruvate dehydrogenase proteins (PDHB, PDHA1), which points towards a higher utilization of glucose for pyruvate synthesis, and its oxidation for energy production. Furthermore, ULU piglets increased the expression of *AQP7*, which codes for an aquaporin with roles carrying out the inflow/outflow of water through the cell membrane, that has been reported to be upregulated by cardiac myoblast cells in response to hyperosmotic stress in vitro^41^. By promoting the breakdown of the cell wall during digestion, ulvan lyase supplementation could increase the availability of minerals/electrolytes, possibly leading to a disruption of osmotic homeostasis. However, in a recent study by our research team, we found that feeding piglets with a different seaweed, *Laminaria digitata*, maintained the circulating levels of electrolytes within reference values for swine^42^. The same happened when feeding piglets with *U. lactuca*^19^. Therefore, the exact reason for the apparent disruption of muscle osmotic homeostasis is unclear in the current study. In turn, ULU piglets increased the abundance of proteasome-related proteins (PSMD2, BRWD1) as well as amino acid metabolism proteins (GOT2, BCAT2) and energy production proteins (ATP5PD, FBP2, IDH3B). Altogether, this seems to suggest that ULU piglets are breaking down endogenous protein, using the resulting amino acids as an energy source to meet their requirements. A similar mechanism has been reported in the muscle of piglets fed with restricted dietary contents of sulphur amino acids, where restricted piglets increased pyruvate oxidation along with oxidative stress response proteins, as well as heat shock proteins, including HSPB1^43^. Yu et al.^44^ have reported that pigs fed with low starch and high NDF and ether extract diets suppress mTOR signalling protein synthesis and increase the ubiquitin–proteasome pathway in the skeletal muscle. In the current study, a dietary factor seems to be causing increased protein degradation in ULU piglets, through proteasome activity. Interestingly, this group also increased the abundance of a protein—E3 ubiquitin-protein ligase (CBL)—which marks proteins for degradation via ubiquitination^45^Following this, the proteasome degrades these proteins, generating oligopeptides suitable for hydrolysis, thus contributing to amino acid recycling^46^. These become seemingly available for the action of GOT2 and BCAT2, which convert aspartate and branch-chain amino acids into oxaloacetate^47^ and branch-chain keto acids^48^, respectively. These can then be used in the TCA cycle further downstream, where proteins such as IDH3B participate. This is rather interesting given that Conde-Aguilera et al.^43^ have reported that a lack of amino acid balance leads to fattier carcasses and IDH3B has furthermore been related to fat-accumulation in pigs^49^. The disruption of muscle metabolism homeostasis is additionally supported by the higher abundance of oxidative metabolism proteins (CAT, GSTM3, GLRX3) and heat shock proteins (HSP90, HSPB1 and HSPA1A) in ULU muscle. As mentioned, these piglets increased the expression of *AQP7*, likely in response to osmotic stress. Furthermore, piglets fed with seaweeds are also being fed with more iodine and bromine, which can accumulate in the muscle^6^ and have the potential to disrupt nutrient metabolism, as well as increase oxidative stress^50^. Therefore, the precise cause for these changes is most likely multi-factorial.

The last comparison, ULU vs UL, returned the lowest number of either DEG’s or DAP’s. Piglets fed with *U. lactuca* without enzymatic supplementation upregulated *HBEGF*, which codes for heparin-binding epidermal growth factor, reported to increase protein synthesis rates in bovine cells^51^. Conversely, ulvan lyase supplementation upregulated the expression of *SMTNL1* and *RXRG*, which respectively participate in muscle contraction^52^ and has been found to be upregulated in the adipose tissue of Iberian pigs fed with high contents of oleic acid^53^. *RXRG* has been reported to be activated by long-chain fatty acids, thereby increasing the transcription of PPAR (proliferator-activated receptors), which signals fatty acid oxidation^54^. This could suggest higher fatty acid oxidation in the muscle of piglets supplemented with ulvan lyase. Nonetheless, we did find an increased abundance of glutathione S-transferase (GST3) and catalase (CAT) in the muscle proteome of ULU compared to UL. Interestingly, higher expression of PPARs has been related to increased catalase expression and/or activity^55,56^, demonstrating that *RXRG* might have a regulatory role in oxidative stress response. Like in previously mentioned instances, the precise reason for this seemingly increased oxidative stress caused by seaweed, is not clear. However, given the fact that ulvan lyase degrades the cell wall of the seaweed during digestion, it is possibly related to the higher availability of intracellular micronutrients for digestion/absorption, like the already mentioned iodine. The fact that ULU piglets increased the abundance of heat shock proteins (HSPB1 and HSPA1A) compared to UL, indicating disrupted homeostasis, further supports such a possibility.

To find associations between transcriptomics and proteomics, we have carried out data integration of both differentially abundant/expressed datasets to find the top 10 most relevant features, similar to previously reported approaches^25,57^. We found that a group of five proteins (GOT2, COL1A2, IRF8, DGKH and LRP5) were negatively related to *PER2* and positively related to *NAV2*, *YPEL2* and *ARNTL* genes. Interestingly, Cardoso et al.^58^ have reported an opposite relation between *PER2* and *ARNTL* (two genes related to the circadian rhythm) expression in the muscle of pigs slaughtered at different time points after being fed, and found this differential expression to be related to different availability of nutrients. The negative feedback between these two genes was later confirmed in a separate study by the same team^59^, where *ARNTL* and *PER2* were down and upregulated, respectively, by sows with lower nutrient availability. In the current analysis, the two genes were found to have relations with proteins involved in nutrient metabolism. The GOT2 gene, which participates in amino acid metabolism, has been reported to be downregulated in the hypothalamus of pigs fed three different meals per day, compared to pigs fed *ad libitum*^60^. In turn, diacylglycerol kinase (DGKH) has been related to cattle growth, due to its role in the cell growth promoting Ras/Raf/MEK/ERK signalling pathway^61^. Accordingly, LRP5 has been reported to promote cell proliferation^62^. Lastly, COL1A2, as well as other collagen proteins that make up the extracellular matrix, is highly expressed in the muscle of pigs that accumulate high contents of intramuscular fat^63^. These abundance differences suggest that ULU piglets have higher nutrient availability in the muscle, leading to a higher abundance of proteins involved in nutrient metabolism and/or cell growth, which is counterintuitive since control should have increased nutrient availability. It is also important to take into consideration that correlation does not necessarily mean causation. Nevertheless, this analysis demonstrates the effect of these diets on circadian metabolism and nutrient utilization in the muscle.

**Figure 8:**
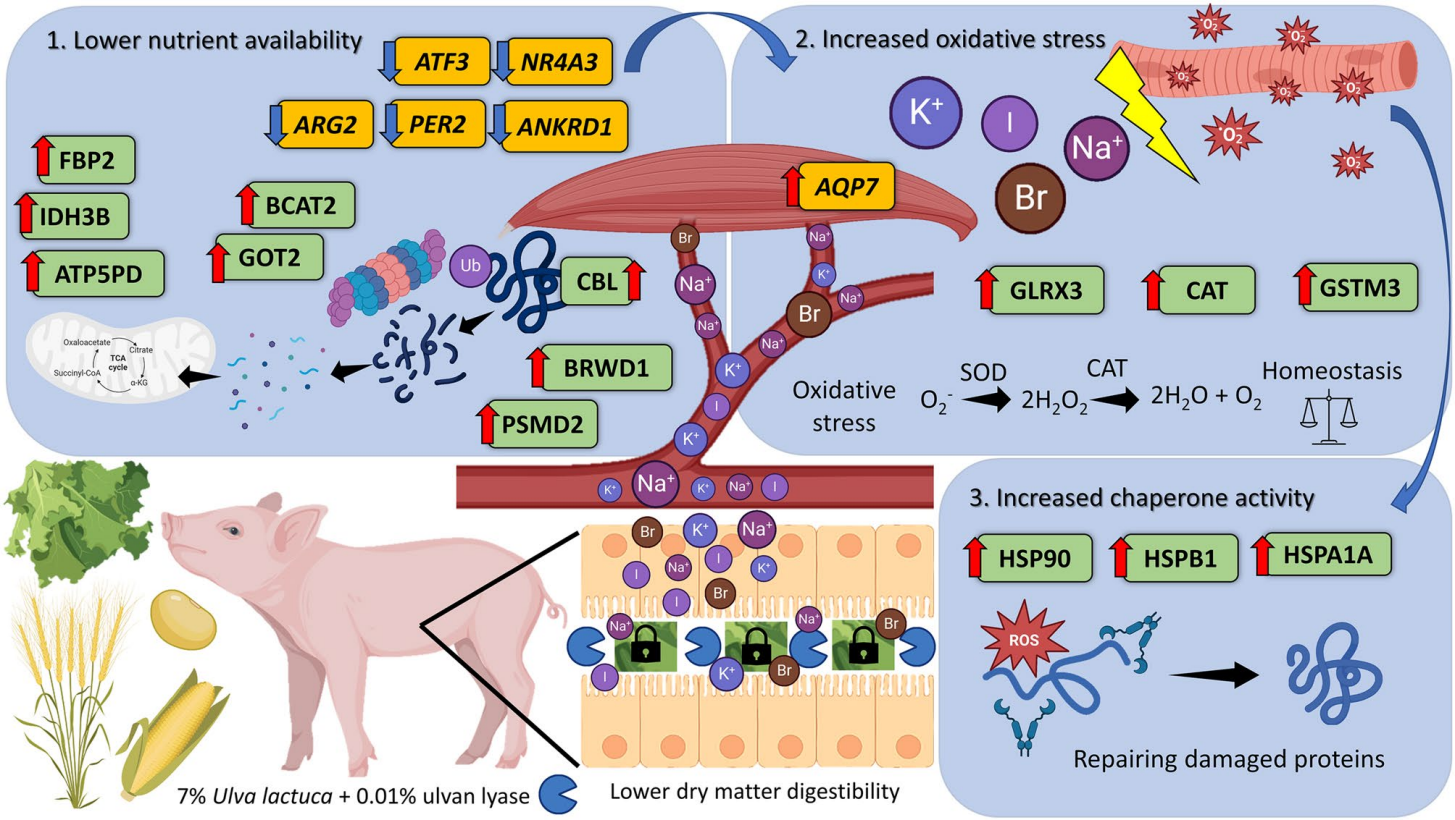
Summary of the interpretation of the differentially abundant genes and proteins of ULU compared with control, depicting the interrelationship between reduced nutrient availability, endogenous nutrient mobilization, and the consequent increase of oxidative stress and chaperone activity. Red and blue arrows indicate up and down-regulation of genes (yellow) and proteins (green) in ULU compared to control. SOD—superoxide dismutase; CAT—catalase; IDH3B—Isocitrate dehydrogenase [NAD] subunit; ATP5PD—ATP synthase subunit d; GOT2—Aspartate aminotransferase; BRWD1—Proteasome assembly chaperone 1; GLRX3—Glutaredoxin 3; GSTM3—Glutathione S-transferase; HSP90—Heat shock protein HSP 90-beta; HSPB1—heat shock 27 kDa protein; HSPA1A—heat shock 70 kDa protein 1A; FBP2—Fructose-bisphosphatase; BCAT2—Branched-chain-amino-acid aminotransferase; CBL—E3 ubiquitin-protein ligase CBL; PSMD2—26S proteasome non-ATPase regulatory subunit 2; *ATF3*—activating transcription factor 3; *NR4A3*—nuclear receptor subfamily 4 group A member 3; *ARG2*—arginase 2; *PER2*—period circadian regulator 2; *ANKRD1*—ankyrin repeat domain 1; *AQP7*—aquaporin 7. Figure created using BioRender.com.

Overall, and as summarized in Fig. 8, the reported effects of seaweed dietary inclusion seemingly stem from different nutrient availability. Such lower nutrient availability seems to have caused a degradation of endogenous protein to use amino acids as a source for energy production, as indicated by the higher abundance of PSM2, a proteasomal protein, along with amino acid metabolism proteins (*e.g.* BCAT). This, combined with the muscle accumulation of iodine, bromine, and higher availability of minerals obtained through the seaweed diets, possibly contributed to increased oxidative stress, given the higher abundance of antioxidative proteins such as catalase (CAT), to maintain tissue homeostasis. This osmotic stress is supported by the higher expression of *AQP7*, compared to control. Because oxidative stress damages cellular proteins, the piglets increase the abundance of heat shock proteins (*e.g.* HSP90), whose functions include re-folding damaged proteins. It is noteworthy to point out that some of these proteins were also found to be highly abundant in the UL vs control comparison. However, the higher number of proteins belonging to commonly affected pathways (proteins such as CBL, BCAT2, FBP2, were not identified in UL vs control) and the overall higher number of DAPs and DEGs seems to point towards an added effect of the enzyme. Enzymatic supplementation seems, therefore, to have worsened the baseline effect of feeding the seaweed alone, as a result of degrading the cell wall of *U. lactuca*. This is similar to what our research team has previously reported for Spirulina, where cell wall-targeted enzymatic supplementation reduced crude protein digestibility due to increased digesta viscosity^64^. However, this interpretation is context-dependent and requires further studies to confirm the generated hypothesis.

## Conclusions and future perspectives

We found that feeding piglets with *U. lactuca* and ulvan lyase supplementation may have reduced macronutrient availability, leading to protein degradation through the proteasome pathway and utilization of amino acids as an energy source. The accumulation of elements, such as iodine and bromine, may have increased oxidative stress in muscle tissue. To counterbalance this stress, anti-oxidative stress proteins, including catalase, were upregulated. The upregulation of the AQP7 gene, associated with osmotic stress response, supported tissue homeostasis maintenance. Chaperone activity, exemplified by HSP90, played a crucial role in protein repair. Overall, our results indicate that ulvan lyase supplementation may exacerbate the effects of *U. lactuca* feeding, possibly due to cell wall degradation during digestion.

Further research is needed to validate these findings in industry-like conditions and explore other omics approaches, such as metabolomics. Examining additional tissues and profiling the accumulation of heavy metals in piglet tissues would provide insights into their contribution to oxidative stress and metabolic changes.

## Materials and methods

### Animal ethics statement

All experimental procedures were approved by the School of Agriculture (ISA) Ethics Commission, as well as national authorities (Direção Geral de Alimentação e Veterinária), under process number 0421/000/000/2020, in compliance with European Union legislation (2010/63/EU Directive) and ARRIVE guidelines 2.0 (https://arriveguidelines.org/arrive-guidelines).

### Live animal trial

The live animal trial took place at the experimental facilities of the Animal Production Department of the School of Agriculture (Instituto Superior de Agronomia) of the University of Lisbon, Portugal. The experimental conditions have already been described elsewhere and are briefly mentioned here for contextual purposes, including the chemical composition of the diets^19^. Briefly, thirty male piglets (Large White × Duroc, aged 28 days) were randomly distributed into three experimental diets (n = 10): control (standard wheat, maize, soybean meal diet), UL (7% *Ulva lactuca* replacing the control diet) and ULU (UL + 0.01% ulvan lyase). They were individually housed in metabolic cages with free access to water, in the framework of a metabolic trial. After 5 days of adaptation, the trial lasted two weeks, during which piglets were daily fed on a pair-feeding basis and weighed at the beginning and end of each week. At the end of the trial, all piglets were slaughtered and the *longissimus lumborum* was sampled, split into aliquots and frozen at − 80 °C until proteomics analysis. For transcriptomics analysis, muscle tissue samples were rinsed in DNA/RNA Shield® reagent (Zymo Research, Irvine, CA, USA) and stored at − 20 °C until RNA extraction.

### Transcriptomics

The transcriptomics analysis has been described previously. For a more detailed description, please refer to our earlier work^25^. Briefly, RNA was isolated from muscle samples of 6 randomly chosen piglets from control, UL and ULU groups. Muscle RNA was extracted and purified using TRIzol (Invitrogen, Carlsbad, CA, USA) and RNeasy mini kit (Qiagen, Hilden, Germany), respectively. Sample DNA was then digested with a treatment of DNAse I (Qiagen, Hilden, Germany), following the manufacturer’s instructions. RNA quantification was done using a spectrophotometer (Nanodrop ND-2000c, NanoDrop, Thermo Fisher Scientific, Willmington, DE, USA). Quality control was carried out on a Fragment Analyser (Agilent Technologies, Santa Clara, CA, USA), using a High Sensitivity RNA Analysis Kit (Agilent Technologies, Santa Clara, CA, USA). cDNA was obtained from 1 µg of RNA samples, using the cDNA Library Construction Kit (Clontech, San Jose, CA, USA), according to the manufacturer’s instructions. RNA libraries were then prepared using adapted protocols^65,66^. Libraries were sequenced on an Illumina NextSeq500 Sequencer (Illumina, San Diego, CA, USA) using a 75 SE high throughput kit. Raw data was analysed using the FastQC tool (https://www.bioinformatics.babraham.ac.uk/projects/fastqc/, accessed 30/03/2023), *fastp* tool^67^ and *hisat2* tool (http://daehwankimlab.github.io/hisat2/, accessed 30/03/2023), using the *Sus scrofa* genome as a reference for read alignment. Gene expression values were quantified using the FeatureCounts program implemented in Subread software (version 1.5.1)^68^. Three comparisons were computed (UL vs control, ULU vs control, and ULU vs UL) using *DESeq2* (version 1.34.0) with an adjusted *P*-value of 0.05 and log_2_(fold change) threshold of − 1 and 1.

### Proteomics

The label-free proteomics experiment was performed by adapting a previously published protocol^69^. For each sample, freeze-dried tissue was homogenized in liquid nitrogen. The homogenate was suspended in trichloroacetic acid—TCA (10%) in cold acetone with 0.1% dithiothreitol—DTT. Each tube was sonicated (Bioruptor plus, Diagenode, Belgium) for 10 cycles (30 s on/off), followed by a 3 h incubation at − 20 °C. Samples were then centrifuged at 15,000 g for 10 min at 4 °C, and the supernatant was discarded. The pellet was washed twice with cold acetone, followed by centrifugation of 3 min at 15,000 g at 4 °C, discarding the supernatant. The pellet was then dried at room temperature (RT) and was resuspended in lysis buffer (urea 7M, thiourea 2M, CHAPS 0.5%), using agitation (1 h, 1000 rpm at RT). Protein quantification was carried out using the RC DC™ (reducing agent and detergent compatible) Protein Assay kit II following the manufacturer’s instructions (Bio-Rad, Hercules, California, USA). Then, 20 µg of total proteins were run on short migration using Criterion XT precast 1D gel 12% Bis–Tris (Bio-Rad, Hercules, California, USA). Two groups of bands of high and low molecular weight were divided and placed in well-plates, where each group was reduced (ammonium bicarbonate NH_4_HCO_3_ 100 mM, DTT 10 mM), alkylated (NH_4_HCO_3_ 100 mM, iodoacetamide—IAA 55 mM) and unstained (NH_4_HCO_3_ 50 mM, 50% methanol—MeOH). Digestion was carried out using trypsin (5 ng/µL, MS grade), incubated overnight at 37 °C. Peptides were extracted acetonitrile—ACN 50%/trifluoroacetic acid—TFA 0.1%, then dried and stored at − 20 °C until LC–MS analysis. For sample injection, the peptides were solubilised with 40 µL of loading buffer (ACN 2%, TFA 0.05%, H_2_O LC–MS).

Peptides were separated using a NanoLC 425 Eksigent System coupled to a TripleTOF 6600 MS system (Sciex, Foster City, CA, USA). Briefly, samples were desalted in a trap column (C18 acclaim™ PepMap™, 5 μm, 5 mm × 300 μm, Thermo Scientific, Bremen, Germany) for 5 min at a flow rate of 2 μL/min using a loading buffer (2% v/v acetonitrile, 0.05% (v/v) trifluoroacetic acid). Then, peptides were separated onto a C18 reverse phase column at a flow rate of 300 nL/min (C18 acclaim PepMap 100, 3 μm, 100 Å, 75 μm × 15 cm, Thermo Scientific, Bremen, Germany) using a gradient (solvent A: H_2_O LC–MS, 0.1% (v/v) formic acid; solvent B: acetonitrile, 0.1% (v/v) formic acid). Peptides were eluted from 3% B to 30% over 60 min, increased to 40% B for 10 min then increased to 80% B until 10 min to wash the column, and then re-equilibrated before the next injection for 20 min at 3% B. An MS survey scan from 300 to 1250 m/z with 250 ms of accumulation time was followed by 30 MS/MS scans (mass range 100–1500 m/z) using the automatically adjusted system of rolling collision energy voltage. Acquired MS and MS–MS data were imported into Progenesis QIP software (version 4.2, Nonlinear Dynamics, Waters, Newcastle upon Tyne, UK). The protein and peptide identification searching the *Sus scrofa* database on UniprotKB (189,471 sequences; 93,216,990 residues, downloaded on 7th September 2021) via Mascot Daemon (version 2.8.0. Matrix Science, London, UK) were imported to Progenesis QIP and matched to peptide spectra. The following Mascot research parameters were used: peptide tolerance of 20 ppm, fragment mass tolerance of 0.3 Da, up to two missed cleavages, carbamidomethylation of cysteine as fixed modification and methionine oxidation, N-terminal protein acetylation and tryptophan to kynurenine as variable modifications. Only the proteins identified with a significant Mascot calculated confidence of 95% were accepted.

### Data analysis

For transcriptomics, a multi-dimensional scaling analysis (MDS) plot, a hierarchical clustering and a volcano plot were generated using the R software, with the *ggplots2* package. Proteomics data were quantitatively and qualitatively analysed. Protein identifications were considered if at least two peptides and one unique peptide were identified per protein. Differentially abundant proteins (DAPs) were considered when ANOVA *P*-value < 0.05 and fold change (X vs Y) was higher than 1.5 (higher in X) and lower than 0.67 (higher in Y). Principal components analysis (PCA) was carried out for the identified proteins using the *fviz_pca_ind* function of the *FactoMineR* package; and a heatmap analysis was performed using the *heatmap.2* functions of the *gplots* package; in the RStudio (v. 3.6.2) software. A Venn diagram was done using an available online tool to depict common DAPs between comparisons (https://bioinformatics.psb.ugent.be/webtools/Venn/, accessed 16/03/2023). Functional analysis was carried out using the ClueGO plugin (v. 2.5.9.)^70^ of the Cytoscape (v. 3.8.2.) software^71^ for each comparison, using a right-sided hypergeometric enrichment and a Bonferroni *P*-value correction. The differentially abundant genes and proteins were used as input for an integrated analysis using the DIABLO method^72^ of the *mixOmics* package^73^ in the RStudio environment. Data from both platforms was centred (column means were subtracted from observations) and scaled (centred values are divided by the standard deviation of the column). The design matrix was established with 0.9 and the number of components to use was obtained using the *perf* function, with Mfold validation. The 10 most significant features were selected from each dataset (5 from each component). The correlation circle plot and circos plot were obtained using the *plotVar* and *circosPlot* functions, respectively.

### Supplementary Information

Supplementary Figure S1.

Supplementary Table S1.

Supplementary Table S2.

## Data Availability

The datasets generated during the current study are available from the corresponding author upon reasonable request. Proteomics raw data has been submitted to ProteomeXchange Consortium^74^ via the PRIDE^75^ partner repository with the dataset identifier PXD043394. RNA-seq raw data was submitted to the Sequence Read Archive (SRA) database under accession PRJNA1056470 and in the Gene Expression Omnibus (GEO) under the identifier GSE252125.
