# Supplementary material for: Integrated transcriptomics and proteomics analysis reveals muscle metabolism effects of dietary Ulva lactuca and ulvan lyase supplementation in weaned piglets
Source: Sci Rep. 2024 Feb 26;14:4589. doi: 10.1038/s41598-024-55462-2 (PMC10967369; doi:10.1038/s41598-024-55462-2)
Supplement: Supplementary file 1 — Supplementary Figure S1. [file 41598_2024_55462_MOESM1_ESM.docx]

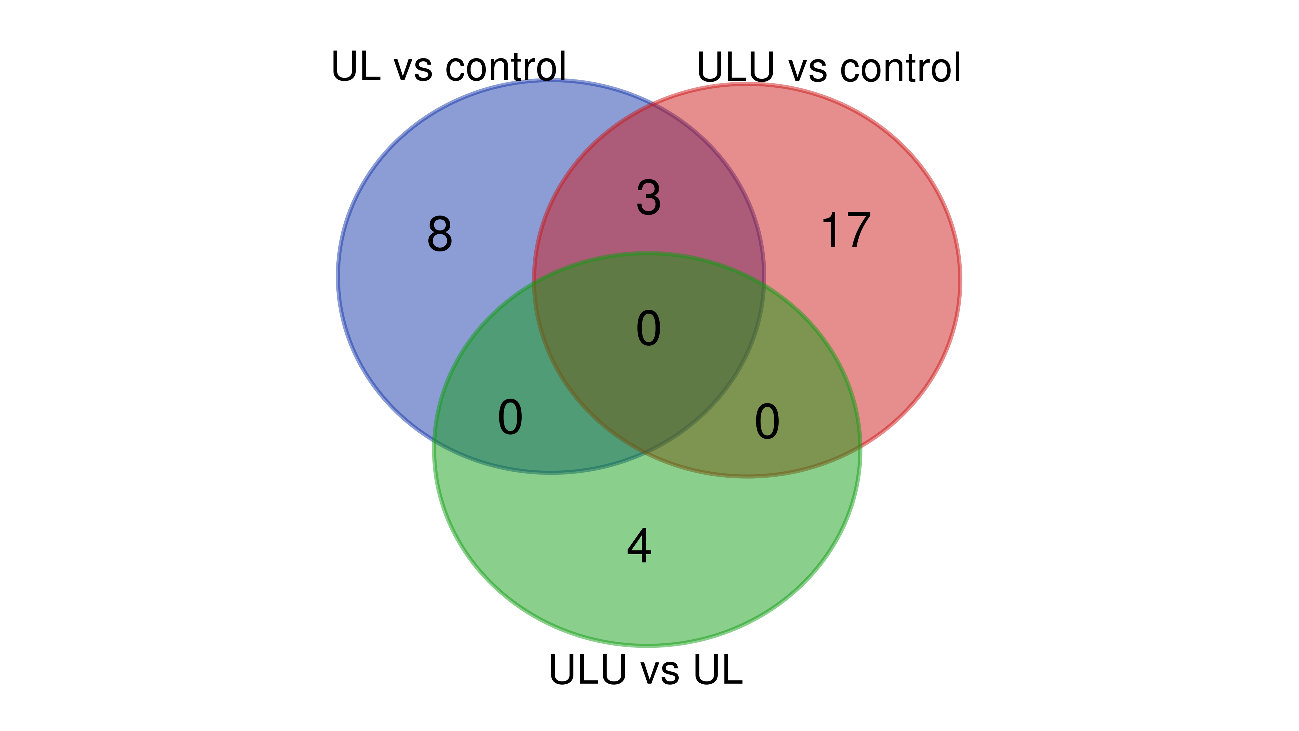


**Supplementary Figure S1** – Venn diagram of differentially expressed genes between each comparison of control (wheat, maize, soybean meal-based diet), UL (7% *Ulva lactuca* replacing control) and ULU (UL+0.01% ulvan lyase).
